# Draft Genome Sequence of Cupriavidus pauculus Strain KF709, a Biphenyl-Utilizing Bacterium Isolated from Biphenyl-Contaminated Soil

**DOI:** 10.1128/genomeA.00222-15

**Published:** 2015-03-26

**Authors:** Takahito Watanabe, Atsushi Yamazoe, Akira Hosoyama, Hidehiko Fujihara, Hikaru Suenaga, Jun Hirose, Taiki Futagami, Masatoshi Goto, Nobutada Kimura, Kensuke Furukawa

**Affiliations:** a Research Institute for Sustainable Humanosphere, Kyoto University, Uji, Kyoto, Japan; b Biological Resource Center, National Institute of Technology and Evaluation (NITE), Shibuya-ku, Tokyo, Japan; c Department of Food and Fermentation, Faculty of Food and Nutrition, Beppu University, Beppu, Oita, Japan; d Bioproduction Research Institute, National Institute of Advanced Industrial Science and Technology (AIST), Tsukuba, Ibaraki, Japan; e Faculty of Engineering, Department of Applied Chemistry, University of Miyazaki, Miyazaki, Japan; f Education and Research Center for Fermentation Studies, Faculty of Agriculture, Kagoshima University, Kagoshima, Japan; g Laboratory of Future Creation Microbiology, Faculty of Agriculture, Kyushu University, Fukuoka, Japan

## Abstract

We report the draft genome sequence of Cupriavidus pauculus strain KF709, which comprises 6,826,799 bp with 6,272 coding sequences. The strain KF709 utilizes biphenyl and degrades low-chlorinated biphenyls; however, it possesses fewer coding sequences involved in the degradation of aromatic compounds than other strains belonging to the *Betaproteobacteria*.

## GENOME ANNOUNCEMENT

Cupriavidus pauculus strain KF709 (NBRC 110672) is one of the 14 biphenyl-utilizing bacteria, termed KF strains, which were isolated from biphenyl-contaminated soil in Kitakyushu, Japan (1). This strain grows on biphenyl; however, it poorly degrades polychlorinated biphenyls (PCBs) compared with other KF strains (1, 2). Southern blot analysis showed that the structures of biphenyl-catabolic (*bph*) genes of KF709 are different from those of the well-characterized Pseudomonas pseudoalcaligenes KF707 and other KF strains (2, 3). Therefore, the genome information of the strain KF709 will shed light on the diversity of biphenyl-utilizing bacteria.

For the strain KF709, a whole-genome shotgun approach was applied using a combined method of shotgun sequencing on a Roche 454 GS FLX+ system and paired-end sequencing on a HiSeq sequencing system (Illumina). The Newbler version 2.6 software package (Roche) was used for the genome assembly. The draft genome size was 6,826,799 bp, containing 227 contigs with an average contig length of 30,074 bp, a median coverage depth of 83-fold, and an average G+C content of 64.0 mol%.

The genome sequence was uploaded to the RAST server (4). A comparison of C. pauculus KF709 with other bacteria within the RAST server identified a nitrogen-fixing bacterium, Cupriavidus taiwanensis (Genome ID 164546.7), as its closest neighbor with a score of 514 (5), followed by a heavy metal-resistant strain, Cupriavidus metallidurans CH34 (Genome ID 266264.4), with a score of 489 (6), and a versatile pollutant degrader, Cupriavidus necator JMP314 (Genome ID 264198.3), with a score of 469 (7). In addition, a biphenyl/PCB-degrader, Burkholderia xenovorans LB400 (Genome IDs 266265.5 and 36873.1), was nominated as a closer neighbor with a score of 143 (8); however, no other KF strains were nominated as closer neighbors. Rapid genome annotation using the RAST annotation server (4) described 6,272 coding sequences (CDSs) and 56 structural RNAs. CDSs were classified into 495 subsystems. It should be noted that the subsystem feature count of the metabolism of aromatic compounds (*n* = 116 CDSs) is less than those of C. taiwanensis (*n* = 148) and C. metallidurans CH34 (*n* = 159), and is also less than half compared with those of C. necator JMP314 (*n* = 327) and B. xenovorans LB400 (Genome ID 36873.1) (*n* = 343).

The strains from the genera Cupriavidus and Burkholderia, belonging to the *Betaproteobacteria*, possess a multipartite genome, and their broad capabilities, such as xenobiotic degradation, chemolithoautotrophy, and symbiotic nitrogen fixation, possibly reflect the acquisition of foreign (ancestral) genes (7, 8). The genome of the strain KF709 revealed that it possesses *bph* genes; however, it exhibits much fewer similarities with those of the strain KF707. Therefore, as a unique Cupriavidus strain, its genome information will provide knowledge as to how multipartite genomes can be formed, how aromatic compound-catabolic genes were acquired, and how the genomic rearrangement occurred through the comparative genomics of betaproteobacteria and the other KF strains.

### Nucleotide sequence accession numbers.

The nucleotide sequence of C. pauculus KF709 has been deposited in the DDBJ/EMBL/GenBank databases under the accession numbers BBQN01000001 through BBQN01000227.
